# Editorial: Finding new epigenomics and epigenetics biomarkers for complex diseases and significant developmental events with machine learning methods, Volume II

**DOI:** 10.3389/fgene.2022.1098821

**Published:** 2022-11-30

**Authors:** Yudong Cai, Peilin Jia, Tao Huang

**Affiliations:** ^1^ School of Life Sciences, Shanghai University, Shanghai, China; ^2^ Beijing Institute of Genomics, Beijing, China; ^3^ Bio-Med Big Data Center, CAS Key Laboratory of Computational Biology, Shanghai Institute of Nutrition and Health, Chinese Academy of Sciences, Shanghai, China

**Keywords:** machine learning, NGS-next generation sequencing, big data, hereditary diseases, cancer

Next-generation sequencing (NGS) has revolutionized biomedical research, enabling genome-wide screening of genetic defects. As genomic data increases, it will be a challenge to identify genetic patterns with traditional sampling-based statistical methods. Therefore, advanced machine learning methods, such as deep learning, and Artificial Intelligence (AI), can be very beneficial.

In the first volume, we gathered insights on the difference on the multi-omics scale between lung adenocarcinoma (LUAD) and squamous cell lung carcinoma (SCLC), the underlying molecular perturbations and their phenotypic impact in patients with the broad spectrum of intellectual disability (ID), the miRNA expression profiles and clinical data of esophageal carcinoma (EC) patients, the environment of Glioblastoma (GBM) tumor revealed by single-cell sequencing, the methylation and gene expression patterns of atrial fibrillation, the latent disease-lncRNA association prediction (FRMCLDA), the Molecular Prognostic Indicators in Cirrhosis (MPIC) database, the probability matrix factorization (PMFMDA) for discovering potential disease-related miRNAs.

With this volume II Research Topic, we aim to build on the progress demonstrated in the first volume. We hope to gather application of novel interpretable classification algorithms in clinical medicine, multi-omics big data integration analysis for genetic diseases, disease gene identification based on network analysis, eQTL associations between SNPs and genes, optimization theory based on targeted therapy for cancer, development of new NGS based tests for genetic diseases, heterogeneous network construction of disease, genes, proteins, and drugs.

We believe that the machine learning methods will be more and more widely used in clinic, help mining the complex biomedical big data and reveal the big value hidden behind the big data.

